# Skin-on-a-Chip Technology for Testing Transdermal Drug Delivery—Starting Points and Recent Developments

**DOI:** 10.3390/pharmaceutics13111852

**Published:** 2021-11-03

**Authors:** Zsófia Varga-Medveczky, Dorottya Kocsis, Márton Bese Naszlady, Katalin Fónagy, Franciska Erdő

**Affiliations:** Faculty of Information Technology and Bionics, Pázmány Péter Catholic University, Práter u. 50a, H-1083 Budapest, Hungary; varga-medveczky.zsofia@itk.ppke.hu (Z.V.-M.); kocsis.dorottya@itk.ppke.hu (D.K.); naszlady.marton.bese@itk.ppke.hu (M.B.N.); fonagy.katalin@ppke.hu (K.F.)

**Keywords:** topical drug diffusion, skin-on-a-chip, microfluidics, Franz diffusion cells, skin equivalents, drug delivery

## Abstract

During the last decades, several technologies were developed for testing drug delivery through the dermal barrier. Investigation of drug penetration across the skin can be important in topical pharmaceutical formulations and also in cosmeto-science. The state-of- the-art in the field of skin diffusion measurements, different devices, and diffusion platforms used, are summarized in the introductory part of this review. Then the methodologies applied at Pázmány Péter Catholic University are shown in detail. The main testing platforms (Franz diffusion cells, skin-on-a-chip devices) and the major scientific projects (P-glycoprotein interaction in the skin; new skin equivalents for diffusion purposes) are also presented in one section. The main achievements of our research are briefly summarized: (1) new skin-on-a-chip microfluidic devices were validated as tools for drug penetration studies for the skin; (2) P-glycoprotein transport has an absorptive orientation in the skin; (3) skin samples cannot be used for transporter interaction studies after freezing and thawing; (4) penetration of hydrophilic model drugs is lower in aged than in young skin; (5) mechanical sensitization is needed for excised rodent and pig skins for drug absorption measurements. Our validated skin-on-a-chip platform is available for other research groups to use for testing and for utilizing it for different purposes.

## 1. State of-the-Art in Testing Topical Drug Absorption and Delivery

Transdermal drug delivery has a high importance as an alternative to traditional routes of drug administration (e.g., per os and intravenous). It means that noninvasive drug administration and transdermal drugs are able to bypass the liver first pass metabolism and reduce the likelihood of side effects due to lower systemic exposure.

Topical drug administration and drug formulation has had a renaissance during the last decades. The target organ of the topically applied drugs can be the skin itself, but several other therapeutic indications are also possible (pain, inflammation, central nervous system effects, humoral effects, etc.), which can be treated by transdermal drug delivery. Testing the drug penetration through the dermal barrier is an important task in the development of new drug formulations in pharmacokinetic/pharmacodynamic (PK/PD) profiling studies and in dermatology. In addition to cosmeto-scientific research, there is a need to test the transcutaneous absorption of active and cosmetic ingredients. To fully utilize the potential of the topical administration route, it is important to optimize the delivery of active ingredients/drugs into or through the skin. The optimization of carrier/vehicle composition is very important at the early phases of product development. A rational approach in designing and optimizing skin formulations requires well-defined skin models, which are able to identify and evaluate the intrinsic properties of the formulation. Most of the current optimization methods rely on the use of suitable ex vivo animal/human models. However, increasing restrictions in the use and handling of animals and human skin stimulated the research for suitable artificial skin models as well.

Currently, as a first approach, in silico studies have been performed as a substitute of in vivo testing to simulate the drug absorption through the skin in the early phase. In silico physiological modeling is critical for predicting dermal exposure to therapeutics and assessing the impact of different formulations on transdermal distribution [1]. VeriSIM Life (VSL) has developed BIOiSIM, a dynamic, biology-driven model which provides a scalable computational solution through the use of machine learning (ML) integrated physiological modeling to make fast predictions that can be applied to larger compound datasets. Integration of ML with mechanistic modeling allows BIOiSIM to complete biological datasets [1].

**Figure 1:**
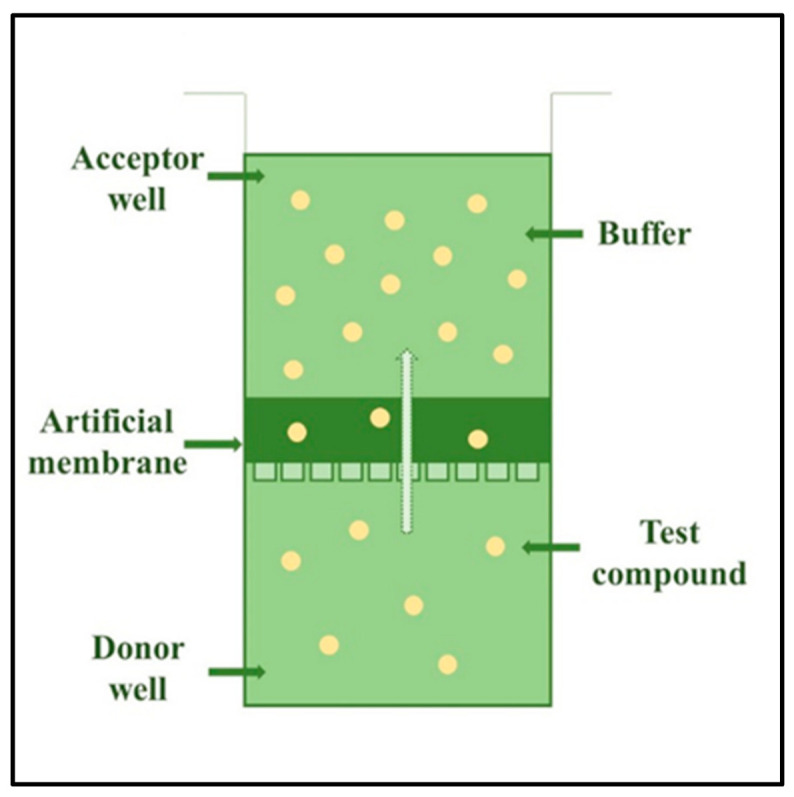
The structure of one-well of a 96-well plate of a parallel artificial membrane permeability assay (PAMPA) system.

**Figure 2:**
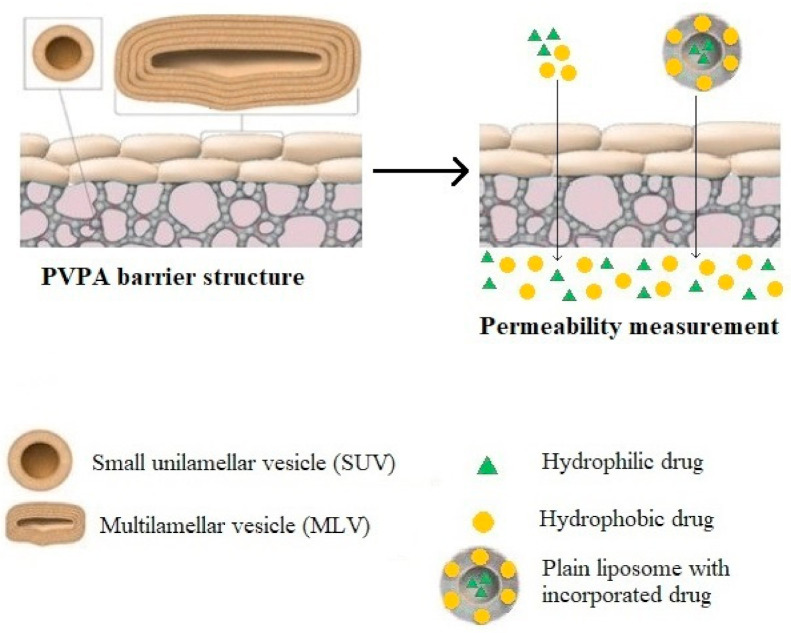
The phospholipid vesicle-based permeation assay (PVPA), mimicking the stratum corneum. It consists of a tight barrier of liposomes deposited on a cellular ester filter support.

The next step in the complexity order of testing platforms is the use of in vitro skin models. There exist different model membranes for testing drug permeability: (1) non-lipid based membranes such as silicone membranes and the Strat-M™ membrane, which is composed of multiple layers of polyether sulfone, creating a morphology similar to human skin, including a very tight surface layer (stratum corneum) and (2) lipid-based model membranes, e.g., parallel artificial membrane permeability assay/PAMPA/and phospholipid vesicle-based permeation assay/PVPA/etc.) [2]. The structure of the PAMPA and PVPA systems are shown in Figure 1 and Figure 2.

In vitro skin cell culturing has greatly developed over the last decades and it is now a well-established technology in drug testing. Although many models have been designed to assess tissue responses to the application of irritants or upon wound healing, the absence of immune cells limits their physiological relevance, underlining the need for more advanced models that better mimic human physiological responses that would lead to the replacement of animal models. In vivo, the skin response to inflammation not only involves tissue-resident cells, but also a range of immune cells that are recruited after pro-inflammatory signals, which are released at the site of injury in the skin [3]. Depending on the application, different cell types have been included in skin cultures (keratinocytes, fibroblasts, melanocytes, macrophages, etc.), however, the inclusion strategies, scaffolds, cell sources, culture media, and culture times are highly heterogeneous [4,5,6]. Keratinocyte cultures serve as a skin model cultivated with collagen in different types of scaffolds. HaCaT cells are immortalized human keratinocytes and have been extensively used to study the epidermal homeostasis and pathophysiology [7].

To make more reproducible models, reconstructed human epidermis or full thickness skin models have been developed. As cell-based techniques, these skin models are useful tools for testing the phototoxicity, corrosivity, and irritancy, as well as drug permeability. Recently, the models were also utilized in the optimization of vehicle composition of different topical formulations.

## 2. Diffusion Studies

For the assessment of topical drug absorption through the skin, different technical solutions (devices and equipment) are available. Additionally, for drug penetration testing, various transport surface materials and biological systems have been developed. The different methodological arrangements have advantages and weaknesses. The right selection of the sufficient model is determined by the question to be answered. This can be related to the number of compounds/formulations to be investigated, the amount of the test substances and vehicle available, the dimensions of the diffusion surface materials (membrane, cell culture monolayer, excised skin) and the time factor. In addition, the costs can be considered as a determining factor in the decision. In the following Section 2.1 and Section 2.2, the most widely used technologies, diffusion platforms, and objects are discussed.

### 2.1. The Testing Platforms—Technologies

#### 2.1.1. Diffusion Cells

One of the most common methodologies for studying transdermal drug delivery in vitro or ex vivo are the diffusion cell systems. The first equipment was developed and described by Thomas J. Franz [8]—however, nowadays several subgroups can be distinguished based on the different parameters such as the orientation (horizontal vs. vertical), geometry, and volume of the compartments, or dynamics of the fluid flow in the receptor chamber (static vs. flow-through).

In general, the diffusion cells consist of a donor and a receptor compartment, separated by a barrier, i.e., an artificial membrane, cell culture, or skin sample. In the static diffusion cells, the receptor chamber solution is continuously stirred with a magnetic bar to provide the uniform distribution of the penetrated substance. The solution is sampled and replaced with a new receptor fluid at each time point, which can be performed either manually or automatically. The μFLUX™ diffusion cell (Pion Inc., Billerica, MA, USA) is a horizontal diffusion cell system where the compartments are divided by a synthetic membrane. It is mostly applied for studying the permeation and dissolution of poorly water soluble drug candidates [9]. A further horizontal set-up is the Side-Bi-Side™ (PermeGear, Hellertown, PA, USA) diffusion cell system, which is suitable for studying the blood-brain barrier or the nasal pathway [10]. A special chamber has been developed for imitating the surface of the cornea, as well. Moreover, the real-time impedance measurement is also possible in both chambers. The Navicyte Horizontal Diffusion Chamber System (Warner Instruments, Holliston, MA, USA) is usually used for studying toxicology or transport mechanisms in interfaces which are exposed to air (for instance dermal, pulmonary, corneal, or nasal cells) [11]. The vertical arrangement of the Navicyte system has also been developed, which is primarily designed for work with excised tissue segments and mostly used for the characterization of the intestinal, corneal, or nasal permeability [12,13,14]. The vertically oriented Franz diffusion cell system (Teledyne Hanson Research, Chatsworth, CA, USA) is one of the “gold-standard” methods for the investigation of the transdermal drug delivery, which is suitable for studying artificial membranes and ex vivo skin samples [15,16]. Beside the transdermal route, several examples are known for Franz cell experiments, studying the nasal, corneal as well as the transbuccal administration [10,17,18,19].

The other group of diffusion cells are the “flow-through” cells, which require a pump to ensure the continuous fluid flow through the receptor chamber. It maintains the sink conditions during the experiment, which is beneficial in the case of drugs having large permeability coefficients [20], moreover this feature simulates the in vivo conditions better than the static mode. The In-Line Cells from PermeGear (Hellertown, PA, USA) are flow-through vertical diffusion cell systems available on the market. Various types of experiments using in-line cells can be found in the literature, such as examination of synthetic membranes mimicking human skin, ex vivo animal, and human skin or even nail samples or buccal membranes [21,22,23]. A vertical, flow-through diffusion cell system has also been developed by our research group, which is described in details in Section 4. The different diffusion cell types are summarized in Table 1.

#### 2.1.2. Organ-on-a-Chip Systems

Predicting the pharmacokinetic and dynamic properties of the candidate drugs is resource- and time-consuming, moreover the translational possibilities of the 2D cell culture and animal models are limited.

A novel, alternative approach is provided by the so-called organ-on-a-chip systems. It utilizes the advanced techniques of microfabrication and tissue engineering, which also enables to eliminate the discrepancy between the human and animal organs, as well as the problems of the low availability of human samples. These systems overcome the problem of the homogenous cell cultures, since the complex interactions of cell and tissue types can be reproduced.

Moreover, tissue barriers, parenchymal tissues, and interorgan interactions can be mimicked as well [27]. There are organ-on-a-chip methods for imitating the lung, heart, liver, kidney, intestine, muscle and placenta [27,28]. Models showing both physiological and non-physiological conditions can be designed, which are proper disease models, such as various tumors [27] or skin diseases described in Section 5 as well.

The ultimate goal is to fabricate a “body-on-a-chip” device, which includes multiple microscale cellular environments simulating the systematic function of a human organism. Such a kind of chip could be used to study a complex pharmacokinetic profile of drugs, covering the absorption, distribution, metabolism and excretion. However, the proper, physiologically relevant scaling of the different organs remain a challenge, moreover the cost of manufacturing is relatively high [27,28].

Currently there is only a few examples for the application of different drug formulations in skin-on-a-chip systems [29,30]. The most used forms are creams and gels, but other solid, semisolid or liquid formulations can also be used in such devices. For a summary of conventional and novel topical drug dosage forms that can be tested in skin-on-a-chip device, see Table 2 and Table 3. [31].

### 2.2. The Diffusion Objects (Diffusion Surfaces)

#### 2.2.1. In Vitro Cell Culture Models

A huge milestone was achieved in the field of skin models by Rheinwald and Green in 1975. They used lethally irradiated 3T3 fibroblasts as feeder layers to create cultures of human keratinocyte colonies from keratinocyte from human skin biopsy. This discovery made it possible for the scientists to generate large quantities of keratinocytes for in vitro cell culture studies and gave a perspective for the treatment of burning injury patients [32,33,34,35]. Rheinwald and Green were also the first to describe that such monolayer cultures can differentiate and form multilayered structures [36]. Their method was further developed by many research groups, for a recent application see Wufuer et al., 2016 [37]. Simplicity and reproducibility are major advantages of monolayer keratinocyte cultures. However, many features of epidermis are lacking in this model and keratinocytes are forced to adapt to artificial circumstances [38].

#### 2.2.2. Human Reconstructed Tissues Models

In the past 10 years, several tissue culture-based 3D human skin models have been developed and become commercially available [39]. They are usually classified as human reconstructed epidermis models (e.g., EpiSkin, SkinEthic, EpiDerm) and living full thickness skin equivalent models (GraftSkin, EpiDermFT, Pheninon). The models are composed of the human cells grown as the tissue culture and matrix equivalents normally present in the skin [40,41]. These models have numerous application possibilities: skin irritation, corrosion, hydration, drug delivery, anti-aging, UV protection, anti-psoriatic, and anti-melanoma drug diffusion testing, etc. For a summary of the applications of the marketed products, see Table 4 [42,43,44,45,46,47,48].

#### 2.2.3. Ex Vivo Excised Skin Models

Numerous articles reported on the evaluation of skin formulations based on the use of ex vivo models of either human or animal origin. The choice of an appropriate ex vivo model can be influenced by the storage, sample handling, preparation technique, and accurate experimental setup for the evaluation of the drug permeability. The selection of species is also an important factor to be considered. The comparison of some features of rat, mouse, pig, and human skins is summarized is Table 5. The strength and weaknesses of different skin model diffusion objects are summarized in Table 6.

## 3. Franz Diffusion Cell Studies at PPCU

### 3.1. Filter Paper and Artificial Membranes

In our initial pilot experiments, filter paper (Whatman 50) and cellulose-acetate membrane (Sartorius) permeability was tested and compared to rat skin in Franz diffusion cells using caffeine cream as a hydrophilic model drug [50]. The degree of diffusion of the active molecule was proportional with the pore size of the diffusion object (filter paper: 2.7 μm, cellulose acetate membrane 0.45 and 0.8 μm) and the excised ex vivo rat skin permeability was comparable to 0.45 μm membrane. 

### 3.2. Excised Skins of Different Species

The next question to be discussed was the optimization of the degree of mechanical sensitization of the excised skin surface before the permeability experiments. This intervention helps to get higher drug concentrations in the receiver chamber of the diffusion cells to make the detection possible by different analytical techniques. For this purpose, different species (mouse, rat, pig, and human) were investigated and various numbers of tape stripping processes were applied (0, 5, 10, 20, 30). The thinner skins (mouse, rat) needed less sensitization (5, 10) while the thicker skins expected more tape stripping (20, 30) for the proper permeability. Based on the results for the routine topical transport and absorption experiments, 10-fold sensitization was utilized in excised epilated rat skin.

Different topical drug formulations (e.g., erthromycine and Aknemycine creams or quinidine cream and gel) were also compared in Franz diffusion cells. The effect of skin sample freezing and thawing and the role of the age of the excised skins were also tested in some experiments. Both the freezing and the aging (2–3 months young and 16–22 months old rats were compared) influenced the drug absorption in a statistically significant manner. However, the lipophilic character of the test compounds was also a determining factor in this respect in transdermal delivery.

## 4. Skin-on-a-Chip Technology at PPCU

**Figure 3:**
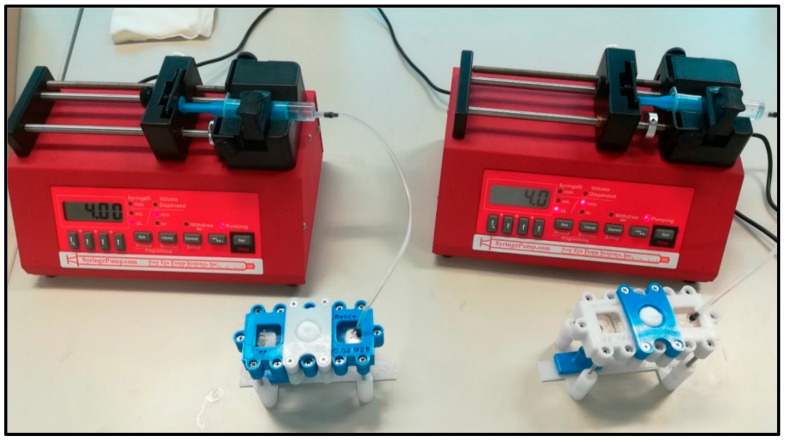
Two simultaneous “skin-on-a chip” experimental setups. The system consists of a programmable syringe pump and a flow-through dynamic microfluidic device [30]. The samples are collected below the diffusion system in the collection bench.

Skin-on-a-chip devices offer innovative and state-of-the-art platforms essential to overcome the limitations of other diffusion cell techniques [51]. In our laboratory, a novel microfluidic device concept has been designed for testing drug penetration through the skin (Figure 3). The generalized design is optimized for manufacturing with rapid prototyping techniques; CNC milling, polymer casting, laser cutting and 3D printing. The developed Microfluidic Diffusion Chamber (MDC) can be used for in vitro/ex vivo monitoring of the transdermal delivery of topical drugs.

The generalized device design consists of three main parts: a polymer-based microfluidic channel assembly, a frame that surrounds the microfluidic channel system and a sample holder that holds and inserts the membrane (diffusion well or integrated skin sample) into the MDC. The device is fabricated using rapid prototyping technologies, which allow us to create multiple MDCs that have slight variations in their design according to the needs of the experiments. Such variations can be the presence or absence of a temperature control unit, the geometry of the microfluidic channel system, the construction of the membrane receiving area, or the configuration of the fluid inlet and outlet ports.

In all device generations, the polymer-based channel assembly is made from polydimethylsiloxane (PDMS) (SYLGARD™ 184 Silicone Elastomer Kit, Dow Consumer Solutions, Los Angeles, CA, USA). The microfluidic channel is created by mixing the PDMS liquid monomer and its treating agent at a 10:1 ratio and incubating it at 70 °C for 2 h in a CNC milled casting mold made from acrylic or aluminum. The polymerized PDMS is cut to shape and punched through at the inlet and outlet ports. If the channel system is created from multiple PDMS parts, then the layers are held together by sandwiching them between laser-cut (Epilog Zing 16 Laser) poly(methyl methacrylate) (PMMA) plates. The microfluidic channel assembly is then inserted into the frame which is held together with bolts and nuts.

### 4.1. The Device (First- and Second-Generations)

The first-generation MDCs were designed and manufactured by Lukács et al. [29]. The chip was designed to be used with skin samples that are stretched at the bottom of the membrane holder. In this design, the membrane holder is merged with the donor compartment, creating a box-like shape that is conical in the inside. The examined substance (e.g., a cream) can be loaded into the cone-shaped funnel where at the bottom it is separated from the peripheral perfusion fluid (PPF) filled channels with the skin sample acting as the membrane. This generation of the device used a holder and frame made from polylactic acid (PLA) using 3D printing (CraftBot Plus 3D printer, CraftUnique Ltd., Budapest, Hungary).

A variation of this device included a combined heater-thermistor unit that could be used for the temperature control of the MDC. The heater unit was placed directly beneath the PDMS layers to ensure good thermal conductivity.

In some experiments, the PLA-based clamping device turned out to not be durable enough, the tensile strength and heat resistance of the PLA material was not sufficient; and the frame cracked under the stress. In these cases, the PLA material was substituted by polyethylene terephthalate glycol (PETG), which has better mechanical properties in this application.

**Figure 4:**
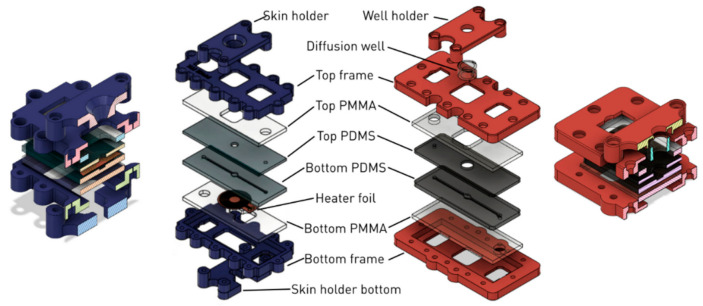
Exploded schematic view of the Microfluidic Diffusion Chamber (MDC) generations. **Left** (blue): Cross-sectional and layer-by-layer view of the first-generation temperature-controlled device with the skin sample holder. **Right** (red): Layer-by-layer and cross-sectional view of the second-generation device with a diffusion well holder.

The second generation was developed to correct the design flaws of the first generation as well as to implement a new sample insertion feature. The second-generation MDCs support two types of clamping devices, the one seen in the first generation and a new one that can be used for the insertion of transwell inserts into the chamber. The wells are placed directly onto the PDMS gel through a window in the acrylic sheet and then the well is pressed down using the well holder (Figure 4).

The frames of the second-generation MDCs were made from PETG or Onyx, the latter is a micro carbon fiber filled nylon material with a very good tensile strength and heat resistance, manufactured by Markforged, Inc., Watertown, MA, U.S.A. Parts created from Onyx were printed on a Mark Two 3D printer (Markforged, Inc.). In some applications the frame and holder were further reinforced with embedded fiberglass or Kevlar (aramid) fibers.

## 5. Drug Penetration Studies

During drug development, in addition to the development of the appropriate drug formulation, the penetration of a given drug is studied according to strict regulations (e.g., OECD, Guidance Notes on Dermal Absorption (No. 156) etc. [52,53,54]). Diffusion cells are most commonly used to study the penetration of drugs through the skin, many variants of which are known and available on the market (e.g., open, closed, vertical, horizontal cell, etc.) [55] (see also Table 1), but during the last years, skin-on-a-chip devices are also increasingly used for drug penetration studies [56]. The great advantages of these devices is that they mimic the skin microcircular perfusion by dynamic arrangement, they are simple to use and small in size (less tissue, cell, test substance, and vehicle demand).

At the laboratory of PPCU, drug penetration studies are performed with microfluidic chips, the structure of which is presented in details in Section 4.1. The topical drug absorption studies were preceded by device validation. In our experiments, caffeine was used as a hydrophilic model drug. Caffeine is widely used in topical formulations and is popular in the cosmetic industry for its beneficial effects on skin microcirculation and hair growth [50]. Moreover, caffeine is easily available, inexpensive, and measurable with a UV-VIS spectrophotometer at 273 nm. During validation assays, cut-to-size, properly prepared filter paper, or cellulose acetate membranes of various pore size were placed in the middle compartment of the device [50,57], and then caffeine cream (cream composition described in Lukács, 2019 [29]) was placed in the microchip donor compartment with a Micromen device (positive replacement pipette). Peripheral perfusion fluid (PPF) was passed through the system at a rate of 4 µL/min. Samples were collected and immediately placed onto dry ice and stored at −75 °C until the analytical investigation. The caffeine concentration of the samples was examined with a NanoDrop spectrophotometer. The intra- and inter-device differences were evaluated and compared. A good reproducibility was observed in the repeated experiments. The validation experiments were summarized in the BSc thesis of Lilla Friedreich [57].

In the first-generation skin-on-a-chip devices, the penetration of various drugs (e.g., caffeine, erythromycin, quinidine) was examined in mouse, rat and human excised skin samples. The differences between the species on freshly excised and frozen samples, as well as on native and mechanically sensitized samples (various numbers of adhesive tape-stripping) were investigated [29]. In addition, young and old rat excised skin samples were also examined (both functionally and morphologically) in fresh and frozen samples, thus studying the changes that take place during the process of aging [30]. These studies were recently expanded to include the investigation of pathological skin samples in collaboration with the research group of Prof. Rolland Gyulai at University of Pécs. In these experiments, the drug permeability properties of imiquimod-induced psoriatic mouse skin samples were examined relative to samples from healthy animals and vehicle-treated controls (unpublished data). Our drug penetration studies with Franz diffusion cells as well as the skin-on-a-chip experiments were complemented by various microscopic techniques (scanning electronmicroscopy, two-photon microscopy) to analyze the morphology and structure of the uppermost layer and cross-sectional view of stratum corneum.

## 6. Efflux Transporter Interaction Studies (Fresh and Frozen Tissue, Erythromycin, Quinidine)

The transdermal delivery of efflux transporter substrates has also been examined using the Franz-diffusion cell and the MDC system, which are described in detail in our previous paper [30].

P-glycoprotein (P-gp, MDR1, ABCB1) is an extremely widespread transporter protein, it is expressed in almost all eukaryotic cell (including the different cell types of the human skin), and in parallel its substrate specificity is also exceedingly broad. We could demonstrate that P-gp is functional in the skin in vivo and ex vivo, moreover its transport mechanism contributes to the transdermal absorption. 

In this project, the transdermal penetration profile of two P-glycoprotein substrates, erythromycin and quinidine, was investigated in the presence and absence of a P-gp inhibitor (PSC-833). The achievements showed the inhibitory effect of PSC-833 on the absorption of both tested compounds in the Franz diffusion cells and the MDC system. However, these effects were present only on freshly prepared skin samples, since the protein was damaged during the freezing and thawing of the skin samples, which increased the tissue permeability. Therefore, the use of frozen tissues is not recommended when studying the dermal barrier. 

A further examined aspect is the effect of aging on the transporter function. It is known from the literature that the thickness and the water, collagen, and extracellular matrix contents of the aged skin are reduced, which might be the reason of the measured higher permeability rate of the aged skin for lipophilic drugs and lower permeability for special hydrophilic components.

**Figure 5:**
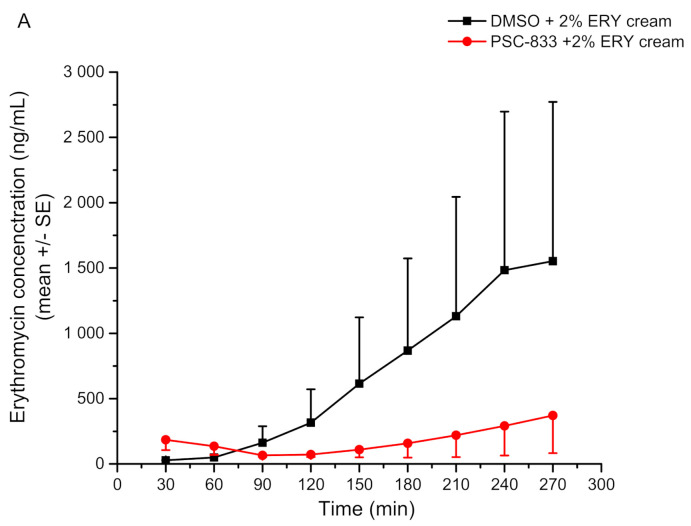

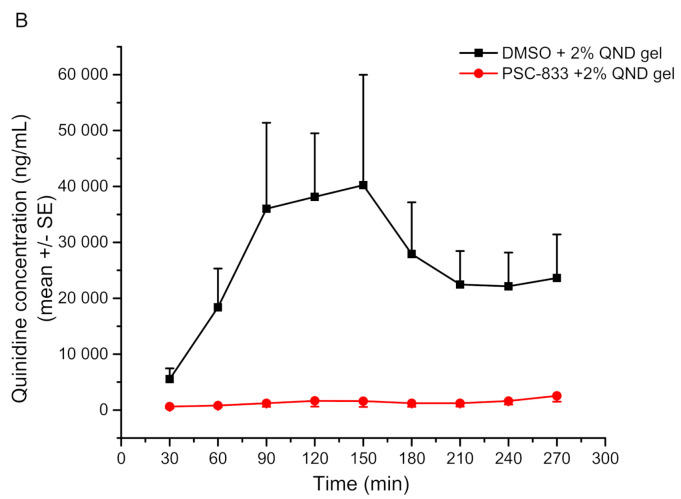
Skin penetration of erythromycin cream (ERY) and quinidine gel (QND) (Panel (**A**,**B**), respectively) across the freshly prepared excised young rat abdominal skin in the microfluidic chip. Concentration data are shown as means +/− SE, *n* = 3–5 [30]. The P-gp inhibitor (PSC-833) significantly reduced QND and ERY penetration through the skin.

It was also demonstrated that the magnitudes of the penetrated substrate concentrations are comparable in studies on the Franz-diffusion cells and on the MDCs, and only the shapes of the curves were different. While they are continuously ascending in the case of the Franz-diffusion cell system, the MDC results have an absorption-plateau-elimination three-phase profile. It corresponds to the differences in the dynamics of the fluid flow of the two systems. As mentioned before, the Franz cell is a static diffusion system, i.e., the penetrated compound is accumulated in the receptor fluid. Although in our 5 h long experiments only the absorption phase could be reached in case of erythromycin cream, when running the experiment for a longer time, after the absorptive period, a plateau phase is outlined (Figure 5A). However, by investigating the quinidine transport from the gel formulation, the three-phase concentration-time profiles have been achieved (Figure 5B). In the MDC system, a third elimination phase is also present because of the flow-through technique, where the drug might be washed out from the receptor compartment. 

In conclusion, it was demonstrated that the MDC system can be utilized for the investigation of transporter interactions at the dermal barrier.

## 7. Skin-Equivalent on a Chip Studies

In recent years, there has been a gradual increase in social pressure to reduce the number of animal experiments. In modern societies, many different stringent laws and regulations have been introduced to meet this demand, including a ban on the use of animals in cosmetic experiments in the European Union since 2013. As a result, there has been a significant increase in the demand for alternatives that can fully elicit the role of animals in pharmaceutical and cosmetic research [58].

**Figure 6:**
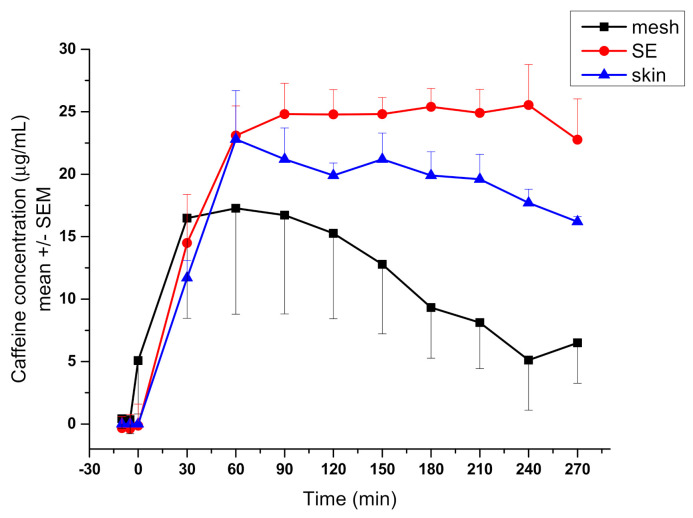
Transdermal transport measurements using skin equivalent (SE) and human skin. Caffeine concentration in the collected fractions was measured by spectrophotometry and is shown as a function of time [58]. The difference between concentration readings from the marked mesh-only and SE samples is significant (*p* = 1.2 × 10^−7^, *n* = 12 in each group).

Skin is a large, heterogeneous, multilayered organ [59]. Due to its complexity, it is difficult to create a proper alternative that can model all the properties of the skin. In collaboration with a research group of Dr. András Czirok at Eötvös Loránd University (Budapest), a measurement system has been created in which the transport of caffeine was successfully examined through a skin equivalent placed in a validated microfluidic chamber. The skin substituent was based on a polycaprolactone (PCL) membrane made by electrospinning [60,61]. Polycaprolactone was used as a scaffold for artificial skin due to its biocompatibility and slow biodegradation [60]. Electrospinning is a versatile technique with a wide range of applications, one of which is biomedical use [61]. The technique makes it possible to produce fibers not only in the micrometer but also in the nanometer range by adjusting the parameters, thereby creating structures that are able to mimic the natural cellular matrix. Our PCL mesh was placed on a 3D printed sample holder consisting of two elements and then the main cylindric element of the sample holder was loaded with collagen-1 gel. HaCaT immortalized human keratinocytes were implanted on the mesh and placed in the sample holder, which attached to the membrane to form a confluent monolayer. During the measurements, the transport of caffeine across the artificial skin equivalent was compared with the human excised skin samples, the results of which showed similar transport kinetics [58] (Figure 6).

## 8. Discussion

As it was demonstrated in the previous paragraphs, the physiological relevance of dermal diffusion models has been improved by various technologies to obtain more accurate and reproducible results in drug and cosmetic research. The current article intended to show some recent advancements in the field of skin-on-a-chip technology achieved at Pázmány Péter Catholic University, a new player of Hungarian medical-biotechnology research. Some details of design, engineering, and manufacturing of the new microfluidic platforms were described and also a few examples of the validation experiments and the major running projects for the utilization of the microchip device were presented. At this phase of the developments, mainly excised human and animal skins and HaCaT cell-based human skin equivalents are used as diffusion platforms. For future directions, more relevant biological platforms are planned to be created and mounted on the chips (e.g., human reconstructed skin and organ substituents with parallelization) [62]. Additionally, organ–organ interactions should be considered at the design and fabrication of novel miniaturized investigational platforms in the pharmaceutical and cosmetical testing laboratories [63].

As the properties of in vitro skin diffusion testing platforms are improving, they have many advantages contrary to the use of in vivo systems. However, they still have some cons as well (Table 7). Therefore, at the selection of the proper permeability model, the purpose of the study and all these aspects need to be considered. 

To further generate more reliable in vitro skin-on-a-chip models, there is a growing interest in integrating different additional skin components, such as microvasculature, immune cells, and hair follicles into the complexity of cell cultures on the diffusion surface of microfluidic devices. On the other hand, some additional factors such as the skin of different populations (pediatric, adult, elderly, different ethnicity, diseased) or different anatomical regions (face, neck, scalp, forehead, upper arm, lower leg, upper leg, inner forearm, outer forearm, back, abdomen, etc.) [64,65] should also be considered when ex vivo models are evaluated. 

These days in silico models greatly help the planning of in vitro/ex vivo testing and contribute to the reduction of in vivo models. On the other hand, the in silico methods are often supported by strong in vitro data and can provide timely results, bringing down costs and the need for extensive biological studies. The in vivo testing can be limited and a large proportion of the required criteria can be planned and met in silico [66,67].

The microfluidic devices and the novel testing technology worked out at Pázmány Péter Catholic University will be further developed, but they are available in the current form for other laboratories and for interested research groups as well.

## Figures and Tables

**Table 1:** Comparison of different diffusion cell types.

Trade Name of the Device	Manufacturer	Structure	Applications	References
μFLUX™	Pion Inc., Billerica, MA, USA	Static, horizontal	Determination of the intrinsic permeability coefficients, dissolution-permeation study of poorly water-soluble candidates through artificial membranes	[9,24]
Side-Bi-Side™	PermeGear, Hellertown, PA, USA	Static, horizontal	Studying the transport mechanism in the blood-brain-barrier and in the cornea, investigation of the nasal dosage forms	[10,25]
Navicyte Horizontal Diffusion Chamber System	Warner Instruments, Holliston, MA, USA	Static, horizontal	Investigation of the toxicology or transport mechanisms in interfaces exposed to air (dermal, pulmonary, corneal, nasal cells)	[10,26]
Navicyte Vertical Diffusion Chamber System	Warner Instruments, Holliston, MA, USA	Static, vertical	Characterization of the intestinal, corneal, or nasal permeability	[12,13,14]
Franz-Diffusion Cell	Teledyne Hanson Research, Chatsworth, CA, USA	Static, vertical	Investigation of the transdermal, nasal, corneal, or transbuccal administration	[10,17,18,19]
In-Line Cells	PermeGear, Hellertown, PA, USA	Flow-through, vertical	Studies for artificial or ex vivo skin or nail samples, or buccal membranes	[21,22,23]

**Table 2:** Different topical dosage forms that can be used in miniaturized diffusion cell device.

Conventional Topical Dosage Forms	
Solids	Powders, plasters
Liquids	Lotions, solutions, suspensions, collodions, liniments, emulsions
Semi-solids	Ointments, creams, pastes, gels, suppositories
Miscellaneous	Transdermal delivery systems, alcohols, medical tapes

**Table 3:** Novel topical dosage forms that can be used in miniaturized diffusion cell device.

Novel Topical Dosage Forms
Novel gels
Aerosol foams
Microsponges
Muco- and bio-adhesives
Novel vesicular carriers
Nano-emulsions and nano-emulgels
Proteins and peptides
Polymers
Emulsifier-free formulations
Fullerenes

**Table 4:** Application possibilities of marketed RHE and LSE products.

Reconstructed Human Epidermis Models (RHE)		
Product	Manufacturer	Main Application Areas
EpiDerm	MatTek Corporation, Ashland, MA, USA	Skin irritation, skin corrosion, skin hydration, dermal drug delivery, phototoxicity, dermal genotoxicity, epidermal differentiation
EpiSkin	L’Oréal, Lyon, France	Skin irritation, skin corrosion, UV protection, bacterial adhesion, DNA damage, dermal drug delivery
SkinEthic	SkinEthic, Lyon, France	Skin irritation, skin corrosion, UV protection, bacterial adhesion, DNA damage, dermal drug delivery, medical devices
SkinEthic RHPE	SkinEthic, Lyon, France	UV exposure, OMICS, depigmentation
EpiCs	CellSystems, Troisdorf, Germany (HENKEL), Phenion	Skin irritation, skin corrosion
EpiCs-M	CellSystems, Troisdorf, Germany (HENKEL), Phenion	Skin irritation, skin corrosion, pigmentation, environmental effects
open source reconstructed epidermis model	Phenion, Düsseldorf, Germany	Skin irritation, skin corrosion
Straticell	Straticell, Les Isnes, Belgium	Skin aging, barrier function, damage related to light, acute inflammation, pigmentation, pollution,
EPI MODEL	Labcyte, Gamagori, Japan	Skin irritation, skin corrosion
**Full-Thickness (living) Human Skin Models (LSE)**		
EpiDermFT	MatTek Corporation, Ashland, MA, USA	Anti-aging, wound healing, skin hydration, UV protection
Phenion Full-Thickness Skin	Phenion, Düsseldorf, Germany	Skin physiology and biochemistry, clinical dermatology, transdermal drug delivery studies, skin penetration studies, wound healing, toxicological assessment of chemicals, analysis of environmental effects on skin physiology, e.g., UV and IR irradiation
Phenion FT-AGED	Phenion, Düsseldorf, Germany	For testing skin aging
GraftSkin	Apligraf; Organogenesis, La Jolla, CA, USA	Wound and injuries, pressure ulcers, varicose ulcers, etc.
Vitrolife-Skin	Kyoto, Japan	Skin irritation, skin corrosion

**Table 5:** Comparison of the dermal barrier properties of four different species (modified from Liu et al., 2009 [49]).

	Mouse	Rat	Porcine	Human
**Full skin thickness (average)**	0.4–1 mm	1–2 mm	1.5–2 mm	2–3 mm
**Epidermal thickness**	9.4–13.3 μm	21.7 μm	52–100 μm	50–100 μm
**Stratum corneum**	2.9 μm	5 μm	12.28 μm	10–12.5 μm
**Fixed skin**	no	no	yes	yes
**Average hair density**	658 hairs/cm^2^	289 hairs/cm^2^	11 hairs/cm^2^	11 hairs/cm^2^

**Table 6:** Comparison of strengths and weaknesses of different in vitro and ex vivo skin models used in permeability studies (modified from Flaten et al., 2015 [2]).

In Vitro/Ex Vivo Skin Models	Strengths	Weaknesses
Silicone membranes	Low cost No storage problems Reproducible	Non-lipid based Not good model of stratum corneum Non-biological origin
PAMPA	Low cost Storage for longer time Reproducible High throughput	Synthetic lipids or non-lipid based Not good model of stratum corneum Non-biological origin
PVPA	Relatively low cost Storage for longer time Reproducible Lipid composition can be modified Parallelization	Lipid structure is not characterized Non-biological origin
Skin cell cultures	Cell lines or primary cultures are available, Monolayers and multiple layers can be developed	High permeability Not sufficient barrier function High costs, different pH
Reconstructed human skin equivalents	Good reproducibility Wide spectrum of applications	High permeability Not sufficient barrier function High costs, different pH
Ex vivo skins		
Snake	Single animal provides repeated sheds Multiple samples from one shed Storage at room temperature	No hair follicles Not relevant skin metabolism No living epidermis and dermis
Mouse	Easy handling Convenient size Hairless species also available	Very thin skin, High permeability High density of hair follicles Hair removal results in damage Ethical issues, storage issues Storage issues
Rat	Easy handling Convenient size Hairless species also available	Thin skin, high permeability High density of hair follicles Hair removal results in damage Ethical issues Storage issues
Pig	Ears are easily obtained, similarity with human skin	Age and the anatomical region of the animal influence the skin thickness, Hair removal results in skin damage Storage issues
Rabbit	Ears are easily obtained, similarity with human skin	High density of hair follicles Hair removal results in damage Ethical issues Storage issues
Guinea pig	Similarity with human skin Hairless species also available	High density of hair follicles Hair removal results in damage Ethical issues Storage issues
Human	The most relevant model	Inter- and intra-individual variability, differences with age, sex, race, origin, anatomical region

**Table 7:** Advantages and limitations of the use of in vitro skin assays contrary to the in vivo models.

In Vitro Skin Permeation Studies	
Pros	Cons
Reduce the number of experimental animals	Physiologically limited relevance
No ethical issues	Lower complexity of the test system
Good reproducibility	Focuses only on one target
High throughput	Not all dermal cell types are included
Relatively fast	No proper circulation
Lower costs than the in vivo	No immunological reactions
Lower standard errors	
Can be more specific	
Mechanistic approach	
Human cells/tissues can be used, high relevance	

## Data Availability

The data of our experiments can be found in the laboratory archive.
